# Establishing MinION Sequencing and Genome Assembly Procedures for the Analysis of the Rooibos (*Aspalathus linearis*) Genome

**DOI:** 10.3390/plants11162156

**Published:** 2022-08-19

**Authors:** Yamkela Mgwatyu, Stephanie Cornelissen, Peter van Heusden, Allison Stander, Mary Ranketse, Uljana Hesse

**Affiliations:** 1 Department of Biotechnology, University of the Western Cape, Robert Sobukwe Road, Bellville 7535, South Africa; 2 Agricultural Research Council, Biotechnology Platform, 100 Old Soutpans Road, Onderstepoort 0110, South Africa; 3 South African Medical Research Council Bioinformatics Unit, South African National Bioinformatics Institute, University of the Western Cape, Robert Sobukwe Road, Bellville 7535, South Africa; 4 Institute for Microbial Biotechnology and Metagenomics, University of the Western Cape, Robert Sobukwe Road, Bellville 7535, South Africa

**Keywords:** rooibos, plant genome assembly, Oxford Nanopore, Canu, MaSuRCA, Haslr, Wengan, Flye, Redbean, Raven, NextDenovo, Racon, Medaka, Nextpolish

## Abstract

While plant genome analysis is gaining speed worldwide, few plant genomes have been sequenced and analyzed on the African continent. Yet, this information holds the potential to transform diverse industries as it unlocks medicinally and industrially relevant biosynthesis pathways for bioprospecting. Considering that South Africa is home to the highly diverse Cape Floristic Region, local establishment of methods for plant genome analysis is essential. Long-read sequencing is becoming standard procedure for plant genome research, as these reads can span repetitive regions of the DNA, substantially facilitating reassembly of a contiguous genome. With the MinION, Oxford Nanopore offers a cost-efficient sequencing method to generate long reads; however, DNA purification protocols must be adapted for each plant species to generate ultra-pure DNA, essential for these analyses. Here, we describe a cost-effective procedure for the extraction and purification of plant DNA and evaluate diverse genome assembly approaches for the reconstruction of the genome of rooibos (*Aspalathus linearis*), an endemic South African medicinal plant widely used for tea production. We discuss the pros and cons of nine tested assembly programs, specifically Redbean and NextDenovo, which generated the most contiguous assemblies, and Flye, which produced an assembly closest to the predicted genome size.

## 1. Introduction

Most large plant genomes contain a high proportion of repetitive DNA as a result of whole-genome, chromosomal, subchromosomal and/or tandem duplications [1,2,3]. These structural features impede whole-genome assembly when using only sequencing data that was produced on Second Generation Sequencing platforms, such as Illumina and Ion Torrent. While accuracy is very high, these sequencers only generate short reads (50–350 bp in length), which generally do not span longer repeat regions, leading to incomplete or highly fragmented genome assemblies [4]. Third Generation Sequencing technologies such as Pacific Biosciences (PacBio) and Oxford Nanopore Technologies (ONT) have revolutionized the field of plant genomics, as they produce reads that can be many thousands of base pairs long [5]. Such reads can span large repetitive regions and thus improve the contiguity and quality of genome assemblies. ONT uses a novel nanopore technology to determine nucleotide sequences. Briefly, DNA molecules are pulled through electrically charged nano-scale pores (nanopores). Only one DNA molecule can pass through the nanopore at a time. As the DNA molecule moves through the pore, each nucleotide causes a characteristic disruption in the ionic current. These electrical signals can be visualized in a “squiggle plot” (usually a fast5 file) and are then translated into DNA sequences using deep learning algorithms such as Guppy and Bonito [6].

Nanopore sequencing has gained popularity since its first introduction to the market in 2014 because it surpasses PacBio in read length, with some reads reaching more than 1 Mbp in length [7]. Unlike other sequencers, nanopore sequencing does not require imaging devices and/or bulky sequencing machinery. One of its devices is the MinION MK1B, which weighs less than 100 g and can fit in the palm of a hand. The MinION MK1B can be plugged into a USB port of a standard computer, making it possible to sequence anywhere, even in the field. Additional benefits of MinION sequencing include relatively low costs (a MinION starter pack currently costs USD 1000) and short turnaround times. The main hindrance for nanopore sequencing of plant samples is the requirement of ultra-pure, high molecular weight (HMW) DNA. This is difficult to achieve for plants because they are often rich in secondary metabolites (e.g., polyphenols, polysaccharides), which bind to the DNA during extraction, affecting the quality and quantity of the DNA [8,9]. These impurities block the nanopores, reducing the yields and quality of sequencing runs.

In South Africa, more than 3000 plant species are used in traditional medicine. Medicinally active compounds have been identified from diverse endemic South African plant species, but, so far, close to nothing is known about the genomic background of these plants. Rooibos (*Aspalathus linearis*) is one of the medicinal plant species endemic to the Cape Floristic Region of South Africa. Fermented rooibos leaves are used to make the well-known beverage, rooibos tea, which is exported world-wide. Rooibos tea is low in tannins, high in volatile compounds, caffeine-free and has a diverse profile of phenolic compounds [10]. The main phenolic compounds found in rooibos leaves are aspalathin, PPAG, nothofagin, orientin, isoorientin, vitexin, isovitexin, isoquercetin, quercetin and rutin [11,12]. A growing body of scientific literature provides evidence that these compounds are associated with the diverse medicinal properties of rooibos, including, but not limited to, anti-diabetic, cardioprotective, anti-cancer, anti-ageing and anti-allergic effects [10]. The rooibos-specific C-glucosyl dihydrochalcone aspalathin and the phenylpropenoic acid glucoside (PPAG) are of particular interest, as they are currently being investigated as potential phytopharmaceuticals [13,14]. Rooibos is the first endemic South African medicinal plant for which transcriptome and genome data has been generated using Illumina sequencing technologies [15,16]. K-mer and flow cytometry analyses indicate that the rooibos genome is quite large, between 1 Gbp and 1.24 Gbp [16]. First assembly tests of the rooibos genome using short-read data amounting to at least 235x genome coverage resulted in very fragmented genome assemblies, which is likely due to high contents of repetitive DNA (predicted to be >50% [17]). Contiguity of the assembly could be improved by incorporating MinION data. Here, we report on the local establishment of MinION sequencing procedures, including DNA extraction, DNA purification, library preparation, as well as biocomputational methods for plant genome assembly using short and long-read sequencing data.

## 2. Materials and Methods

### 2.1. DNA Extraction, Purification and Quantification

Leaf material was obtained from one commercial (plant 1) and one wild rooibos plant (plant 2) that originated from Nieuwoudtville, Northern Cape province, South Africa (31°43′17″ S 19°07′29″ E and 31°42’55.0″ S 19°07’40.0″ E, respectively). Plant samples were flash frozen in the field, transported in liquid nitrogen and maintained at −80 °C. DNA was extracted using two methods: a Sodium Dodecyl Sulfate (SDS) method and a hexadecyltrimethylammonium bromide (CTAB) method [18], modified as described below. For all extraction and purification steps, wide-bore tips were used to minimize DNA shearing. The final elution buffer did not contain EDTA, as this may interfere with the construction of MinION libraries. To avoid DNA degradation, samples were stored overnight at −20 °C and sequenced the day after extraction/purification.

For the SDS method, 1 g of rooibos leaf material was ground into a fine powder using liquid nitrogen. Thereafter, 4 mL of heated (55 °C) SDS lysis buffer (containing 20 mM EDTA pH 8.0, 100 mM Tris-HCl pH 8.0, 1.4 M NaCl, 1% (*w*/*v*) SDS, 0.04% (*w*/*v*) PVP-40 and 20 µg/mL proteinase K, to which 0.5% (*v*/*v*) β-mercaptoethanol was added just before use) were poured into the mortar and mixed vigorously as soon as the slur started to thaw. For this protocol, all centrifugation steps were carried out at 8000× *g* for 12 min at room temperature, unless stated otherwise. The homogenate was incubated at 55 °C for 30 min. The solution was centrifuged and the supernatant was transferred to a new tube. This was followed by adding ½ a volume of chloroform, mixing by gentle inversion and centrifugation using a fixed angle rotor. After transferring the supernatant to a new tube, an equal volume of chloroform:isoamyl alcohol (24:1) was added, the solution was centrifuged and the supernatant was recovered. DNA was precipitated by adding 0.1 volumes of 5 M NaCl and 2.5 volumes of 100% ice-cold ethanol, and subsequent incubation on ice for 30 min. If DNA precipitated directly, the DNA was fished out using a hooked glass pipette and transferred to a 1.5 mL microcentrifuge tube containing 100 µL of 10 mM Tris-HCl pH 8.0. If not, the precipitate was centrifuged and the pellet was washed twice with 1 mL ice-cold 70% ethanol. The pellet was air-dried for 5–10 min and dissolved in 100 µL of 10 mM Tris-HCl pH 8.0 at 37 °C for about 1 h.

Thereafter, the SDS samples were purified using chloroform:isoamyl alcohol extraction. For this, an equal volume of chloroform:isoamlyl alcohol (24:1) was added to the sample, the phases were separated by centrifugation and the supernatant was gently pipetted to a new tube. Thereafter, 0.1 volumes of 5 M NaCl and 2.5 volumes of 100% ice-cold ethanol were added and the sample was incubated on ice for 30 min. The solution was again centrifuged and after removing the supernatant the pellet was washed three times with 1 mL of 70% ice-cold ethanol, respectively. The pellet was then air-dried for 5–10 min, resuspended in 100 µL of 10 mM Tris-HCl pH 8.0, and stored at −20 °C until further use.

For the CTAB method, 1 g of rooibos leaves was ground into a fine powder using liquid nitrogen in the presence of 0.5 g PVP (PolyVinyl Pyrrolidone, Mr 10,000), making sure that the tissue never thawed. This was followed by adding 15 mL of freshly prepared CTAB buffer (containing 20 mM EDTA pH 8.0, 100 mM Tris-HCl pH 8.0, 1.5 M NaCl and 2% (*w*/*v*) CTAB, to which 1% (*v*/*v*) β-mercaptoethanol was added just before use). As the slur began to thaw, it was mixed vigorously. The resulting homogenate was transferred into a 50 mL Nalgene tube and incubated at 65 °C for 2 h with intermittent mixing by inversion every 10 min to maximize yields. Thereafter, the solution was centrifuged at 11,000× *g* for 15 min at room temperature and the aqueous phase was carefully transferred into a new 50 mL Nalgene tube. A double volume of chloroform:isoamyl alcohol (24:1) was mixed into the sample by gentle inversion, the tube was centrifuged at 11,000× *g* for 15 min at room temperature and the aqueous layer was recovered. If this aqueous solution appeared translucent, the step was repeated until the solution was transparent. To precipitate the DNA, a double volume of chilled isopropanol and 1/3 volume of 3 M sodium acetate (pH 5.2) were added and mixed by inversion, after which the sample was kept at −20 °C overnight. After centrifugation at 4500× *g* for 90 min at 4 °C, the supernatant was discarded. The pellet was washed twice by adding 5 mL of 70% chilled ethanol, dislodging and gently swirling the pellet, and centrifugation at 4500× *g* for 15 min at 4 °C. The pellet was air-dried, then fully dissolved in 500 µL of nuclease-free water, transferred to a 2 mL Eppendorf tube, re-precipitated and washed as described above, and then dissolved in 100 µL of 10 mM Tris-HCl pH 8.0. Thereafter, the sample was stored at −20 °C, to be used for DNA purification or library preparation.

CTAB-extracted DNA was purified using three commercial kits, namely the Zymoclean™ Large Fragment DNA Recovery Kit (Zymo Research, United States of America), QIAGEN^®^ DNeasy PowerClean CleanUp Kit (QIAGEN, Germany) and QIAGEN^®^ Genomic-tip 500/G (QIAGEN, Germany). When using the Zymoclean™ kit, 3.5 µg of crude rooibos DNA were loaded into one well of a 0.8% agarose gel, which was run at 80 V for 1.5 h. Gel slices with HMW DNA were cut from the gel, further divided into 100 mg cubes, each of which was subsequently processed separately using the Zymoclean™ kit. Both the Zymoclean™ kit and the QIAGEN^®^ DNeasy PowerClean CleanUp kit were used following the manufacturers’ instructions, except that all centrifugation steps were carried out at half the speed and double the time specified in the respective protocols to minimize DNA shearing. For the QIAGEN^®^ Genomic-tip 500/G, we followed two protocols: one published by the manufacture ([19]: Genomic-tip 1) and one published by ONT ([20]: Genomic-tip 2).

DNA concentrations were measured with the Qubit^®^ 2.0 Fluorometer (Thermo Fisher Scientific) using the double-stranded DNA (dsDNA) High-Sensitivity (HS) assay kit (Thermo Fisher Scientific), following the manufacturer’s protocol. The purity was evaluated with a Nanodrop^®^ 2000 (Themo Fisher Scientific), assessing the 260/280 nm and 260/230 nm ratios. The genomic DNA was analyzed on a 0.8% (*w*/*v*) agarose gel: the DNA samples were mixed with 6× purple gel loading dye (New England Biolabs) that had been spiked with red gel (New England Biolabs) in a 1:1 ratio, and the samples were run at 90 V in 1× TBE buffer for 1 h. Lambda DNA digested with *HindIII* was used as a molecular weight marker (New England Biolabs). DNA was visualized using a UV (302 nm) transilluminator and a photo was captured using the ENDURO™ Gel Documentation System.

### 2.2. Genome Sequencing

For the Illumina sequencing dataset, DNA extraction, library preparation and data pre-processing steps have been described previously [16]. Here, the paired-end read datasets were further quality filtered using the bbmap tool FilterByTile (v 37.90, [21]) with default settings. Read quality was subsequently assessed using FastQC (v 0.11.5, [22]) to determine read lengths, read numbers, %GC, and to visualize per tile sequence quality.

Each MinION library was prepared using 1.5 µg of genomic DNA (as measured by the Qubit^®^ Fluorometer) and the ONT 1D Ligation Sequencing Kit, SQK-LSK109 (ONT), following the manufacturer’s instructions. The third-party reagents NEBNext^®^ end repair/dA-tailing Module (NEB #E7546), NEBNext^®^ formalin-fixed paraffin-embedded (FFPE) DNA Repair Mix (NEB #M6630), and NEB Quick Ligation Module (NEB #E6056) were used during library preparation. The adapter-ligated DNA sample was quantified using a Qubit^®^ dsDNA HS assay kit. Each MinION library was sequenced using a FLO-MIN106 R9.4.1 spotON Flow Cell (ONT), which was primed and loaded according to the manufacturer’s instructions on a MinION Mk1B device (ONT). Sequencing was performed for 72 h using the MinKNOW software (v 4.3.20, ONT), installed on a computer running on a Linux operating system (Ubuntu 18.04). The fast5 files containing raw reads were base called using Guppy (v 6.1.7, ONT) with the configuration file dna_r9.4.1_450bps_hac.cfg and default parameters. The read length and quality of the MinION data was assessed using minion_qc (v 1.4.2, [23]) and NanoPlot (v 1.33.0, [24]). Nanoplot computed read statistics using only MinION sequencing reads with a minimum quality threshold of Q ≥ 7.

### 2.3. Genome Assemblies and Evaluation

All programs below were run on a High-Performance Computing cluster at the Centre for High-Performance Computing (CHPC, Cape Town, South Africa).

First, all quality processed Illumina reads (including the MiSEQ and HiSEQ reads from a 300 bp insert library, and the HiSEQ reads from two mate-pair libraries with insert sizes of 3 Kb and 8 Kb, respectively) were assembled using the short-read de-novo genome assembly programs Platanus (v 1.2.4, [25]) and MaSuRCA (v 4.0.1, [26]). The entire dataset amounted to 2.5 billion reads (292 Gbp; minimum 235× genome coverage). For Platanus, the genome size was set to 1.2 Gbp and the k-mer value to 81 (same as the one automatically computed by MaSuRCA). Platanus was run in three distinct steps (contig assembly, scaffold assembly and gap closing), all with default parameters. For MaSuRCA, a configuration file was created, which contained the paths to the input data, as well as the assembly parameters, all set to default. A shell script was then generated from the configuration file to run the final assembly. Two passes of mega-reads were performed.

Hybrid assemblies using 40× of Illumina and 25× of MinION data were performed with MaSuRCA (v 4.0.1) as described above, as well as with Haslr (v 0.8a1, [27]) and Wengan (v 0.2, [28]) using default settings. The data size had to be reduced because hybrid assemblers are resource-intensive.

MinION data amounting to 25× genome coverage was also de-novo assembled using the long-read assemblers Flye (v 2.8.3, [29]), Raven (v 1.6.0, [30]), Redbean (wtdbg2, v 2.5, [31]), Canu (v 2.2, [32]) and NextDenovo (v 2.4.0, [33]). Again, the programs were run with default parameters, setting the genome size to 1.2 Gbp. Long-read assemblies were polished with two long-read and two short-read polishing programs. Briefly, assemblies were first polished with four rounds of Racon (v 1.4.3, [34]) using all long reads and recommended parameters (-m 8 -x 6 -g 8 -w 500), where read mapping was completed using minimap2 (v 2.22, [35]). Next, the Racon consensus was polished with one round of Medaka (v 1.5.0, [36]) using the model r941_min_hac_g507. Medaka was run in three distinct steps, namely: (1) mini_align to align reads to the input assembly; (2) medaka consensus, which runs the consensus algorithm and outputs results into hdf files; and (3) medaka_stitch, which aggregates the results of the previous step and creates a consensus sequence (.fa or fasta). The Medaka-polished consensus was further polished with Illumina reads using one round of either Racon or Nextpolish (v 1.1.0, [37]), all parameters set to default.

Assembly statistics were generated with QUAST-LG (v 5.0.2, [38]) with the following parameters: -e -b -large -glimmer -est-ref-size 1200000000 -no-snps. Genome assembly accuracy and completeness was assessed using BUSCO (v 5.2.2, [39]).

## 3. Results

### 3.1. Optimizing DNA Sample Purity

**Figure 1:**
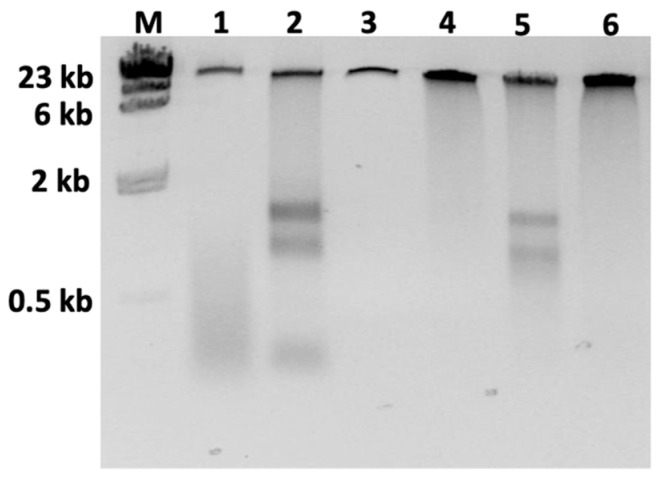
Agarose gel electrophoresis of 120 ng DNA extracted from rooibos leaves using either SDS or CTAB extraction protocols. Lane M: *HindIII* molecular weight marker; Lane 1: SDS, unpurified; Lane 2: CTAB, unpurified; Lane 3: CTAB, ZymocleanTM Large Fragment DNA Recovery Kit; Lane 4: CTAB, QIAGEN^®^ DNeasy PowerClean CleanUp Kit; Lane 5: CTAB, Genomic-tip 1; Lane 6: CTAB, Genomic-tip 2.

Our first goal was to optimize DNA extraction and purification procedures to generate ultra-pure HMW genomic DNA, suitable for long-read sequencing. Of the two tested DNA extraction procedures, the SDS-based method returned 4×–25× less DNA than the CTAB-based protocol (Table 1 and Table S1). In addition, the 260/230 ratios of the SDS samples were outside the range of values associated with high purity of DNA, indicating trace amounts of organic solvents (260/280 and 260/230 ratios should be within 1.8–2.0 and 2.0–2.2, respectively). For the CTAB samples, the 260/280 and 260/230 ratios were within the expected ranges. However, here 3–5-fold differences in DNA concentrations between the Qubit and Nanodrop readings were observed, which implies presence of contaminants. Gel electrophoresis confirmed that both extraction methods yielded intact HMW DNA and revealed a substantial amount of RNA in the CTAB samples (Figure 1, Lane 2). Due to higher yields, only CTAB-extracted DNA samples were used to establish DNA purification procedures.

Four DNA purification methods were tested. Of those, the worst results were obtained when using the Zymoclean™ (Zymo) kit. While Nanodrop absorbance curves for the other three DNA purification methods showed well-defined peaks with a maximum at 260 nm (Figure S1d–f), the samples purified using the Zymo kit showed a maximum absorbance around 235 nm (Figure S1c). Moreover, the 260/280 and 260/230 ratios were out-of-range, and a significant amount of DNA was lost during the clean-up procedure (at most, 600 ng were recovered out of 3.5 µg of crude, CTAB-extracted DNA). When CTAB-extracted DNA samples were purified using the QIAGEN^®^ Genomic-tip 500/G (Genomic-tip), the 260/280 and 260/230 ratios were close to the intended ranges, but the Qubit:Nanodrop ratios were still too high. So, the Genomic-tip 1-purified samples still contained substantial amounts of RNA (Figure 1 Lane 5). Independent of the protocol, only 2–6.9% of the DNA was recovered after Genomic-tip DNA purification. By far the best results were consistently obtained when purifying the crude DNA using the QIAGEN^®^ DNeasy PowerClean CleanUp (DNeasy) kit. The DNA was ultra-pure, as indicated by good absorption ratios and a nearly 1:1 relationship for the Qubit:Nanodrop DNA concentrations. Recovery of HMW DNA ranged between 39% and 70% (Table 1 and Table S1).

### 3.2. Nanopore Sequencing

Table 2 shows the run statistics for seven MinION sequencing runs: run1 was completed using CTAB-extracted unpurified DNA from rooibos plant 1; runs 2–4 using DNA from plant 1 that was purified using different clean-up procedures; run5 using CTAB-extracted DNA from plant 2 purified with the DNeasy kit, and runs 6 and 7 using oxidized leaf samples of plant 1, where the CTAB-extracted DNA was also purified using the DNeasy kit. Each run was conducted on a separate MinION flowcell with 1003–1485 pores available before sequencing, and lasted 72 h, i.e., until nearly all pores were irreversibly exhausted.

The unpurified DNA sample of plant 1 (run1) yielded the least amount of data (1 Gbp). For this sample, sequencing statistics were lowest for all measured parameters, including number of sequenced bases, number of reads, read length, N50 and read quality. Nonetheless, this run generated over 300,000 high-quality reads of up to 58.7 Kbp in length, the total base count amounting to at least 0.8× coverage of the rooibos genome. Samples purified using the Genomic-tip (run2 and run3) generated 4.56 Gbp and 6.62 Gbp of high-quality sequencing data, respectively. Read lengths as well as sequencing quality were substantially improved in comparison to the unpurified sample. The differences between run2 (Genomic-tip 1 protocol) and run3 (Genomic-tip 2 protocol) were not very big: on average, the two runs produced 1.4 ± 0.2 Mio high-quality reads, reaching similar N50s (6.0 Kbp and 6.6 Kbp) and maximum read lengths of 115 Kbp and 75 Kbp, respectively. For plant 1, DNA purification using the DNeasy kit (run4) yielded by far the best results in terms of read numbers (≈5 Mio HQ reads amounting to 19 Gbp), with an N50 of 6.5 Kbp and a maximum read length of 79.6 Kbp. When this clean-up protocol was tested on plant 2 (a wild type of rooibos with high amounts but markedly different profiles of phenolic compounds; run5), the run statistics were again notably higher than for the Genomic-tip-purified samples (3.9 Mio HQ reads, 11.2 Gbp and a maximum read length of 180.9 Kbp). The DNeasy kit was also tested on two oxidized leaf samples of plant 1 (runs 6 and 7). Although all quality tests (light absorbance and Qubit:Nanodrop ratios, as well as gel electrophoresis) indicated that the DNA was of high quality and purity, the total yield in Gbp and the number of reads were much lower than in run4, but still higher than in run1 (the non-purified DNA sample of plant 1). However, the median read lengths and the N50 values were substantially lower than in any of the other sequencing runs.

### 3.3. Evaluation of Genome Assemblies

The second aim of this study was to evaluate different programs for plant genome assembly. Illumina sequencing generated nearly three billion raw reads, with an average read length of 119 bp after removal of adapter sequences. After quality trimming, the data amounted to 235× to 290× genome coverage (depending on whether the flow cytometry or k-mer-derived genome size value is used for reference).

To see how the addition of MinION data improves genome assembly, Illumina data was first assembled using the two de-novo genome assembly programs, Platanus and MaSuRCA (Table 3, Table S2). When using the flow cytometry genome size value (1.24 Gbp) as a reference, the two assemblers reconstructed only 56% (Platanus) and 69% (MaSuRCA) of the rooibos genome into 78 k and 70 k scaffolds, respectively. MaSuRCA outperformed Platanus in terms of scaffold lengths, but assembly accuracy may have been reduced, as indicated by lower BUSCO statistics.

When adding 25× genome coverage of HQ MinION sequencing data from runs 1–4 (amounting to 31 Gbp of data) and 40× genome coverage of Illumina data, the MaSuRCA assembly statistics improved dramatically (Table 3, Table S2): the number of scaffolds was reduced to 29 k, the N50 increased nearly five-fold, the largest scaffold was over 1 Mbp long (15-fold increase), and the proportion of identified BUSCO genes increased to 86% (only 2% of which were fragmented). With approximately 1.5 Gbp, the total assembly length was notably larger than the estimated genome size, indicating incomplete assembly. This was also reflected in the elevated proportion of complete duplicated BUSCO hits (35.3%). Neither Haslr nor Wengan produced reasonable assemblies, as the total assembly lengths (≈0.2 Gbp) were only a fraction of the estimated genome size (Table 3).

The next five tested assemblers (Flye, Canu, Raven, Redbean and NextDenovo) only accept long reads and require polishing of the reconstructed genome using long and short-read sequencing data. The results for the unpolished assemblies differed substantially between the programs (Table 4, Table S2).

Flye generated an assembly closest to the predicted genome size (total length 1.1 Gbp) with the highest proportion of completed BUSCO genes (97.3%). However, the assembly was very fragmented (33 k contigs) and the N50 comparatively low (77 Kbp). Canu produced the same number of contigs and achieved similar BUSCO statistics as Flye, but the total assembly length was below the estimated genome size values. Moreover, the N50 and the largest contig size were the lowest among the long-read assemblers. Raven also generated a comparatively small assembly in terms of total size (0.91 Gbp) and contig numbers (11 k), but BUSCO results indicate high accuracy. With 99 Kbp, the N50 was substantially higher than in the assemblies generated by MaSuRCA, Flye and Canu. The Redbean assembly had the second-highest N50 (142 Kbp) and included a contig of nearly 1.7 Mbp in length. Contig numbers were reduced to half of those generated by MaSuRCA, Flye and Canu. However, BUSCO statistics for the unpolished assembly were the lowest. The most contiguous unpolished assembly was produced by NextDeonovo, which had the highest N50 (218 Kbp) and the longest contig (3.4 Mbp) assembled into just 5431 contigs. However, the total length of this assembly amounted to only 66–82% of the predicted genome size (1–1.24 Gbp).

In general, assembly polishing using long reads only tended to improve assembly statistics, slightly reducing the number of contigs and increasing both the N50 and the maximum contig lengths. While the differences in BUSCO statistics were close to naught for the Flye and Raven assemblies, the proportion of complete BUSCOs in the Canu, Redbean and NextDenovo assemblies increased substantially, with Canu and Redbean assemblies now outperforming Flye and Raven. The two programs used for assembly polishing with the Illumina data (Racon and Nextpolish) produced similar results. This last step of data analysis improved BUSCO statistics even further, the proportion of complete BUSCOs now ranging between 94.5% (NextDenovo assembly) and 99.6% (Canu assembly).

All tested programs required substantial amounts of computational resources (Table 5). Most programs completed analyses when provided with access to 56 cores and a standard 900 GB of RAM, although CPU times differed between 33 h (Medaka polishing) and 2462 h (MaSuRCA hybrid assembly). The exception was Canu, which only completed analyses when provided with 112 cores and nearly 3000 GB of RAM, taking 35,401 CPU hours to run.

## 4. Discussion

Long-read sequencing is becoming standard procedure for plant genome analysis, as these reads are able to span repetitive regions of the DNA, substantially facilitating reassembly of a contiguous genome. With the MinION, Oxford Nanopore offers a comparatively cost-efficient and compact sequencing technology that promises feasibility of high-throughput plant DNA sequencing, even in small laboratories with limited financial means. However, DNA purification and sequencing protocols must still be adapted to generate sufficient high-quality long-read data that would cover larger genomes several times at a reasonable price, particularly when working with non-model plant species.

The main challenge for long-read sequencing of plant DNA using MinION is the requirement of copious amounts of HMW ultra-pure DNA. This is not straightforward, as most plant species contain secondary metabolites that bind to the DNA during extraction, affecting DNA quantity and quality. Rooibos, for example, produces a unique combination of phenolic compounds, including flavones, flavonols and glucosyl dihydrochalcones. The total amount of polyphenols can make up to 30% of the plant dry weight and aspalathin alone can contribute up to 13.5% to the leaf dry weight [40]. In previous studies, we established a DNA extraction procedure that generated high yields of HMW DNA suitable for Illumina sequencing [16]. However, CTAB may be difficult to remove after extraction, and we therefore also tested a DNA extraction protocol that uses SDS as a detergent. When assessing yield and purity of the DNA, the best results were achieved with the CTAB extraction method. Similar results were reported previously in [41], who found that CTAB was more efficient than SDS in extracting genomic DNA from the endemic Brazilian plant species, *Croton linearifolius*. Plants vary dramatically in their chemical compositions, which explains why there is no universal method for plant DNA extraction. CTAB-based methods worked well for diverse medicinal plant species that were high in phenolic compounds [42,43,44], although SDS was the preferred detergent for DNA extraction from eucalyptus [45].

According to [46], the best MinION sequencing results are achieved when the Qubit:Nanodrop ratio for DNA concentrations is close to 1:1, as it implies very high purity of the DNA. Basically, it indicates a high proportion of dsDNA in the sample, which can ligate to the ONT adapter and is then available for sequencing. Neither the SDS nor the CTAB protocol yielded such ultrapure DNA. Since the SDS extraction method did not generate sufficient amounts of DNA, only the CTAB-extracted DNA was further purified using three commercial kits: the Zymoclean™ Large Fragment DNA Recovery Kit, the QIAGEN^®^ DNeasy PowerClean CleanUp Kit and the QIAGEN^®^ Genomic-tip 500/G. The Zymoclean kit failed to generate suitable DNA. It efficiently removed RNA (as visualized on the gel) and even appeared to narrow the Qubit:Nanodrop ratio in comparison to unpurified DNA; however, the 260/280 ratios were always too high and the 260/230 ratios were close to zero. The low 260/230 ratios were most likely associated with the presence of carbohydrates due to insufficient removal of the agarose [47]. This may be the result of reduced centrifugation speeds that were employed to minimize breakage of the DNA. Consequently, this sample was not sequenced. The second-best results were obtained when using the QIAGEN^®^ Genomic-tip 500/G kit, which is the clean-up procedure recommended by ONT (personal communications). In this kit, the columns operate based on gravity flow, which means that centrifugation steps that could potentially damage HMW DNA can be avoided. Of the two tested protocols, the one published by ONT yielded slightly better results in terms of DNA recovery and purity. Nonetheless, the Qubit:Nanodrop ratios in DNA concentration were still high. The QIAGEN^®^ Genomic-tip 500/G protocol had initially been tested using arabidopsis and wheat species [48]. For plant species that are rich in secondary metabolites, such as lavender (*Lavandula angustifolia*), catmint (*Nepeta mussinii*) and poplar species [3,48], additional clean-up procedures (e.g., through Amicon Buffer Exchange or AMPure XP beads purification) were tested with variable results. In our study, by far the best results in terms of DNA purification were consistently obtained when using the QIAGEN^®^ DNeasy PowerClean CleanUp Kit. The DNA losses during purification were comparatively small and DNA purity was excellent, as indicated by the light absorbance and the Qubit:Nanodrop ratios.

When comparing across the DNA purification methods with DNA samples from plant 1, our MinION sequencing results confirm that a close Qubit:Nanodrop ratio is a good indicator for successful sequencing: the best results were obtained when the sample was purified using the QIAGEN^®^ DNeasy PowerClean CleanUp Kit, which yielded 19× more HQ data (in Gbp) than the unpurified DNA, and approximately three times more than the Genomic-tip purified DNA samples at similar N50 values. Good performance of this clean-up procedure was confirmed when using flash-frozen green leaf material of plant 2 (a wild type of rooibos plant that has high concentrations, but substantially different profiles of phenolic compounds). Here, read lengths of up to 230 Kbp were achieved, surpassing the results obtained with the Genomic-tip kit. If not immediately flash-frozen or dried after harvest, rooibos leaves turn brown within a very short period of time, which is associated with the rapid oxidation of the plant material. We therefore also used the QIAGEN^®^ DNeasy kit to purify the DNA from oxidized leaf samples of plant 1, where the DNA had likely undergone some degradation. Even here, the sequencing output of HQ data in terms of total number of nucleotides and maximum read length were comparable to the results obtained with the Genomic-tip kit used on DNA from non-oxidized leaf samples, although read length distributions were substantially skewed toward the smaller size, as indicated by the lower N50 values.

A second challenge for plant genome analysis is the requirement of substantial computational capacities for the reassembly of the genome from the sequencing datasets. Using our Illumina and MinION data, two approaches were tested: hybrid assembly, where long and short reads are used simultaneously, and long-read assembly followed by polishing, first with the long and then with the short-read datasets. When using short-read data only, the hybrid assembler MaSuRCA generated a better assembly than previously tested short-read assembly programs, including ABySS, SoapDenovo and Platanus [17]. The effect of providing MaSuRCA with both, Illumina and MinION data was substantial: total scaffold numbers dropped from 70 k to 29 k, the N50 increased nearly five-fold to 81 Kbp, and the longest scaffold length increased 15-fold to 1.2 Mbp. It must be noted that this program is computationally very demanding: for the combined assembly of the short and long-read data, MaSuRCA required 864 GB and 2462 CPU hours of run time. The other two tested hybrid assemblers, Haslr and Wengan, were found unsuitable for rooibos genome reconstruction. After polishing, several long-read assemblers performed better than MaSuRCA, particularly in BUSCO statistics, which implies better assembly accuracy. Top BUSCO scores were obtained for the Canu, Flye and Redbean assemblies. To date, Canu remains the most commonly used assembler for plant genome analysis [49,50]. It was specifically developed to handle long, high-noise sequencing reads produced by PacBio and MinION. Tests with Macadamia nut [2] and flax [50] datasets showed that Canu outperformed other assembly programs in terms of contiguity and completeness of the reassembled genomes, which is why it was included into the LeafGo protocol for plant genome analysis [51]. In Canu, the first step includes read correction, followed by trimming and assembly. Consequently, this program has substantial computational requirements, which may be a limiting factor when dealing with large heterozygous genomes. Therefore, Canu consistently crashed when attempting to reassemble the highly heterozygous 1.6 Gbp genome of the mollusc *Mytilus coruscus* [52]. In our study, when using only the MinION rooibos sequencing data, Canu also failed to complete the analyses until it was provided with double the number of cores (112) and more than three times the amount of RAM memory (≈3000 GB) than the other programs. Yet, it generated a highly fragmented assembly with a comparatively low N50. This is likely associated with the relatively low genome coverage obtained from the MinION sequencing data (Koren, S.; personal communications). Moreover, our studies indicate that rooibos has a high rate of heterozygosity (approximately 2% [17]), which may have also impeded the assembly. Just like Canu and MaSuRCA, Flye required substantial computational resources and generated a rather fragmented assembly. Previous studies indicated that this assembler is suitable for plant genome assembly [53], but may be less efficient when working with data from highly heterozygous, repeat-rich genomes. This may explain our results, as rooibos is also predicted to have a high proportion of repetitive DNA (>50%; [17]). However, it must be noted that in terms of total assembled sequence length (1.1 Gbp), Flye yielded a value that was closest to the rooibos genome size predicted using flow cytometry (1.24 Gbp). Redbean completed the assembly within 32 h, using only 54 GB of RAM. This program appeared to successfully reconstruct large sections of the genome (as indicated by the second-highest N50 and second-longest contig length), but polishing was found to be essential to improve sequence accuracy. After polishing, however, Redbean yielded outstanding BUSCO values (99.2%), which were comparable to the Canu and Flye assembly results. With 1 Gbp, the total assembly length was similar to the genome size predicted using k-mer analysis (also 1 Gbp). Redbean had performed worst in the Macadamia nut [2] and the flax [50] studies, reconstructing only half of the expected genome sizes. Both plant species have experienced a whole genome duplication event, which permits the conclusion that Redbean may excessively collapse DNA regions with high sequence similarity, leading to higher N50s and longer maximal read lengths, but also to reduced total genome assembly sizes. NextDenovo generated the most contiguous but also the smallest assembly. While the values for N50 and the longest contig length were outstanding, they must be taken with caution. The total assembly may only represent as little as 66–82% of the actual genome size, implying that NextDenovo may have collapsed genome regions with high sequence similarity. In this assembly, a high proportion of BUSCOs were still missing, even after polishing, although not as many as in the MaSuRCA assembly.

## 5. Conclusions

To date, only a few plant genomes have been sequenced using Oxford Nanopore sequencing technologies where the genome size is as big as that of rooibos, or larger [1,54]. Consequently, DNA extraction and purification procedures still require optimization. In this study, DNA purification after CTAB extraction substantially improved yields, particularly when using the QIAGEN^®^ DNeasy PowerClean CleanUp Kit. However, the median read lengths did not improve. Reducing centrifugation speed and size selection before library construction may improve read lengths. Considering the substantially lower price per sample, the QIAGEN^®^ DNeasy kit may be considered a suitable alternative for the QIAGEN^®^ Genomic-tip 500/G kit for DNA purification. With regard to genome assembly, the aim of this study was to obtain an overview on computational requirements and program performance with the rooibos sequencing datasets. Therefore, the different assembly and polishing programs tested here were run using only default parameters, which are more or less optimized for data analysis (see also [55,56,57]). However, each of the programs has a varying number of adjustable multi-level parameters, that permit fine tuning of the assembly and polishing processes. Testing each of these parameters here is beyond the scope of this study. For final genome assembly, the different parameters of the selected program(s) should be optimized. Although Redbean appeared to be the superior assembly program in terms of computational resource requirements and genome assembly statistics, such as N50, contig numbers, assembly length and BUSCO hits, it may potentially collapse regions of high sequence similarity. Future studies will focus on comparative analyses of genome assemblies generated by different assembly programs toward identification of the most accurate representation of the rooibos genome.

## Figures and Tables

**Table 1:** Yields and quality metrics for rooibos DNA samples from two rooibos plants generated using various DNA extraction and purification procedures.

DNA Extraction Method	Plant	Purification Method ^+^	260/280	260/230	Qubit (ng/µL)	Nanodrop (ng/µL)	Q:ND *	Starting Material	Total Yield (µg)	Sequencing Run
SDS	1	C:I extraction	2.12	1.93	20.8	67.5	0.3	1 g leaves	2.0	n/a
CTAB	1	None	2.11	2.01	358	1944	0.2	1 g leaves	35.8	1
CTAB	1	Zymoclean	2.94	0.04	6.3	13.0	0.5	3.5 µg DNA	0.06	n/a
CTAB	1	Genomic-tip 1	2.07	1.91	102	1816	0.1	100 µg DNA	2.0	3
CTAB	1	Genomic-tip 2	2.06	2.22	347	1143	0.3	100 µg DNA	6.9	4
CTAB	1	DNeasy	1.92	2.38	323	337	1.0	10 µg DNA	6.4	2
CTAB	2	DNeasy	1.98	2.35	222	260	0.9	10 µg DNA	4.4	6
CTAB	1	DNeasy	1.92	2.33	325	381	0.9	10 µg DNA	6.5	7
CTAB	1	DNeasy	1.97	2.35	324	336	1.0	10 µg DNA	6.4	8

^+^ Purification procedures include chloroform:isoamyl alcohol (C:I) extraction, the Zymoclean™ Large Fragment DNA Recovery Kit, the QIAGEN^®^ DNeasy PowerClean CleanUp Kit, and the QIAGEN^®^ Genomic-tip 500/G (Genomic-tip 1 and Genomic-tip 2). * Qubit:Nanodrop ratio.

**Table 2:** Sequencing statistics for seven MinION runs with genomic DNA samples from rooibos (Quality ≥ 7).

	Run1	Run2	Run3	Run4	Run5	Run6	Run7
total gigabases (Gbp)	1.01	4.56	6.62	19.08	11.24	4.26	2.24
# reads (×1 M)	0.30	1.21	1.56	4.98	3.91	4.25	2.30
N50 length (bp)	4372	6002	6641	6483	5642	1666	1480
median length (bp)	2656	2519	3027	2496	1607	569	587
max length (bp)	60,435	11,5278	75,142	79,588	180,920	116,028	110,672
median Quality	10	11.1	11.4	11.3	11.2	11.2	11
**Read length distributions:**							
>10 kb	11,270	86,876	136,418	401,054	167,363	10,551	4616
>20 kb	598	13,964	17,790	54,087	6700	460	215
>50 kb	2	91	61	123	2	3	4
>100 kb	0	1	0	0	1	1	2
>200 kb	0	0	0	0	0	0	0
**Top 5 longest reads (bp) and their mean quality score:**							
1	60,435 (9.0)	115,278 (10.0)	75,142 (10.9)	79,588 (8.5)	180,920 (19.6)	116,028 (7.9)	110,672 (14.0)
2	57,561 (8.8)	84,582 (9.8)	71,014 (9.0)	75,403 (11.6)	135,178 (7.1)	64,587 (14.9)	102,055 (14.7)
3	45,472 (10.8)	74,614 (12.7)	69,281 (10.6)	74,146 (13.1)	91,826 (7.9)	61,155 (7.7)	99,741 (6.7)
4	42,770 (9.6)	71,567 (10.0)	65,014 (11.0)	70,447 (9.3)	47,153 (10.2)	44,661 (10.3)	95,441 (14.8)
5	42,761 (9.6)	71,536 (11.0)	64,036 (10.0)	68,961 (10.1)	46,147 (12.4)	39,799 (9.9)	90,125 (14.6)

Run1: plant 1 unpurified DNA; run2: plant 1 Genomic-tip 1; run3: plant 1 Genomic-tip 2; run4: plant 1 DNeasy; run5: plant 2 DNeasy; runs 6 and 7: oxidized plant 1 DNeasy.

**Table 3:** Relevant assembly and BUSCO statistics for the assemblies of the rooibos genome generated using Illumina and MinION sequencing data with short-read and hybrid assembly programs.

Assembly Parameters	Illumina		Illumina + MinION		
	Platanus	MaSuRCA	MaSuRCA	Haslr	Wengan
Number of scaffolds	78,315	70,462	29,263	19,723	12,972
Largest scaffold (bp)	154,059	257,349	1,225,954	77,419	222,008
Total length (Mbp)	693.7	857.9	1482.6	173.6	218.6
N50 length (bp)	10,871	17,142	80,888	10,950	22,199
Complete BUSCOs (%)	56.1	45.5	84.3	67.0	49.4
Complete single BUSCOs (%)	51.8	36.9	49.0	62.7	48.6
Complete duplicated BUSCOs (%)	4.3	5.9	35.3	4.3	0.8
Fragmented BUSCOs (%)	34.5	18.8	2.0	12.9	8.6
Missing BUSCOs (%)	9.4	35.7	13.7	20.1	42.0

**Table 4:** Relevant assembly and BUSCO statistics for the unpolished and polished assemblies of the rooibos genome generated using the five long-read assembly programs: Flye, Canu, Raven, Redbean and NextDenovo.

	Assembly Parameters	Unpolished	Racon_Long	Medaka	Racon_Short	Nextpolish
Flye	Number of contigs	33,346	32,286	32,393	31,921	32,392
	Largest contig (bp)	921,299	925,383	928,488	926,118	925,075
	Total length (Mbp)	1118.5	1107.2	1112.5	1107.4	1110.7
	N50 length (bp)	77,709	78,172	78,358	78,512	78,176
	Complete BUSCOs (%)	97.3	96.5	98.0	99.2	99.2
	Complete single BUSCOs (%)	65.9	71.8	70.2	65.5	64.3
	Complete duplicated BUSCOs (%)	31.4	24.7	27.8	33.7	34.9
	Fragmented BUSCOs (%)	2.4	2.7	1.6	0.8	0.8
	Missing BUSCOs (%)	0.3	0.8	0.4	0.0	0.0
Canu	Number of contigs	33,477	33,351	33,357	33,320	33,356
	Largest contig (bp)	434,840	443,413	444,838	443,863	443,487
	Total length (Mbp)	949.0	965.0	970.8	968.1	969.1
	N50 length (bp)	40,175	41,134	41,324	41,225	41,247
	Complete BUSCOs (%)	96.1	98.8	99.2	99.6	99.6
	Complete single BUSCOs (%)	71.4	72.5	72.5	57.6	56.9
	Complete duplicated BUSCOs (%)	24.7	26.3	26.7	42.0	42.7
	Fragmented BUSCOs (%)	2.4	0.0	0.4	0.0	0.0
	Missing BUSCOs (%)	1.5	1.2	0.4	0.4	0.4
Raven	Number of contigs	11,675	11,674	11,674	11,672	11,674
	Largest contig (bp)	760,847	765,889	767,221	765,347	764,913
	Total length (Mbp)	905.6	909.8	915.2	912.6	913.1
	N50 length (bp)	99,352	99,903	100,315	100,069	100,060
	Complete BUSCOs (%)	96.9	95.7	96.9	97.6	98.0
	Complete single BUSCOs (%)	79.6	76.9	79.6	69.0	69.4
	Complete duplicated BUSCOs (%)	17.3	18.8	17.3	28.6	28.6
	Fragmented BUSCOs (%)	1.2	2.4	1.2	0.8	0.8
	Missing BUSCOs (%)	1.9	1.9	1.9	1.6	1.2
Redbean	Number of contigs	16,753	16,347	16,350	16,275	16,350
	Largest contig (bp)	1,686,036	1,739,027	1,738,674	1,734,655	1,734,062
	Total length (Mbp)	962.0	993.5	996.8	994.0	995.1
	N50 length (bp)	142,771	148,572	148,398	148,157	148,018
	Complete BUSCOs (%)	83.9	96.5	98.8	99.2	99.2
	Complete single BUSCOs (%)	80.4	82.4	85.9	74.9	75.3
	Complete duplicated BUSCOs (%)	3.5	14.1	12.9	24.3	23.9
	Fragmented BUSCOs (%)	8.6	2.7	0.4	0.4	0.4
	Missing BUSCOs (%)	7.5	0.8	0.8	0.4	0.4
NextDenovo	Number of contigs	5431	5431	5431	5431	5431
	Largest contig (bp)	3,406,093	3,359,923	3,371,738	3,361,759	3,361,805
	Total length (Mbp)	824.2	817.1	820.9	818.6	818.9
	N50 length (bp)	218,190	217,600	218,885	218,094	218,285
	Complete BUSCOs (%)	90.2	92.1	93.7	94.5	94.5
	Complete single BUSCOs (%)	72.9	72.5	76.1	69.8	69.8
	Complete duplicated BUSCOs (%)	17.3	19.6	17.6	24.7	24.7
	Fragmented BUSCOs (%)	3.1	1.2	0.4	0.4	0.4
	Missing BUSCOs (%)	6.7	6.7	5.9	5.1	5.1

**Table 5:** Computational requirements for assemblers and assembly polishing algorithms.

Program	Data	Cores	CPU Time (h)	RAM Memory (GB)	Disc Space (GB)
Platanus	Illumina	56	1308	624	5
MaSuRCA	Illumina	56	1420	426	245
Haslr	Illumina + MinION	56	37	39	50
Wengan	Illumina + MinION	56	465	599	37
MaSuRCA	Illumina + MinION	56	2462	864	543
Flye	MinION	56	863	584	205
Raven	MinION	56	44	36	24
Redbean	MinION	56	205	54	16
Canu	MinION	112	35,401	2960	57
NextDenovo	MinION	56	244	804	7.2
Racon_long *	Polishing	56	186 ± 1.5	776 ± 0.0	577 ± 0.0
Medaka *	Polishing	56	33 ± 0.0	106 ± 0.1	40 ± 0.0
Racon_short *	Polishing	56	167 ± 0.3	654 ± 0.0	554 ± 0.0
Nextpolish *	Polishing	56	37 ± 0.5	54 ± 0.0	51 ± 0.0

* Average values for polishing of the Flye, Canu, Raven, Redbean and NextDenovo assemblies.

## Data Availability

The datasets generated and analyzed in this article cannot be submitted to public databases due to the restrictive Biodiversity Legislation of South Africa.

## Supplementary Materials

The following supporting information can be downloaded at: https://www.mdpi.com/article/10.3390/plants11162156/s1, Table S1: Yields and quality metrics for rooibos DNA samples generated using various DNA extraction and purification procedures; Figure S1: Illustration of DNA purity and Nanodrop readings from different DNA extraction and purification procedures. (a) DNA extraction using SDS method followed by chloroform:isoamyl alcohol extraction. (b) DNA extraction using the CTAB protocol with no further purification. (c) CTAB-extracted DNA purified with the Zymoclean™ Large Fragment DNA Recovery Kit. (d) CTAB-extracted DNA purified with the QIAGEN^®^ DNeasy PowerClean CleanUp Kit. (e) CTAB-extracted DNA purified following the Genomic-tip 1 protocol. (f) CTAB-extracted DNA purified following the Genomic-tip 2 protocol; Table S2: Relevant assembly and BUSCO statistics for the assemblies of the rooibos genome generated using Illumina and MinION sequencing data with short, hybrid and long-read assembly programs.
