# Supplementary material for: Establishing MinION Sequencing and Genome Assembly Procedures for the Analysis of the Rooibos (Aspalathus linearis) Genome
Source: Plants (Basel). 2022 Aug 19;11(16):2156. doi: 10.3390/plants11162156 (PMC9416007; doi:10.3390/plants11162156)
Supplement: Supplementary file 1 [file plants-11-02156-s001.zip › plants-1811001 - Table S1 and Figure S1.pdf]

Table S1: Yields and quality metrics for rooibos DNA samples generated using various DNA extraction and purification procedures

DNA Extraction Method	#Reps	Purification Method *	260/280 Ratio	260/230 Ratio	Nanodrop (ng/µl)	Qubit (ng/µl)	Starting Material	Total Yield (µg)	Sequencing Run
SDS	1	C:I extraction	2.12	1.93	67.5	20.8	1g leaves	2.0	n/a
	2	C:I extraction	2.15	1.90	87.24	33.4	1g leaves	3.3	n/a
	3	C:I extraction	1.97	2.37	39.2	15.0	1g leaves	1.5	n/a
	4	C:I extraction	1.83	0.86	286.51	97.4	1g leaves	9.7	n/a
CTAB	1	None	2.11	2.01	1943.8	358.0	1g leaves	35.8	1
	2	None	2.08	1.88	1106.08	362	1g leaves	36.2	n/a
	3	None	2.10	1.81	1004.69	346	1g leaves	34.6	n/a
	4	None	2.16	1.97	2021.09	380	1g leaves	38.0	n/a
CTAB	1	Zymo	4.53	0.03	11.9	5.5	1mg gel slice	0.544	n/a
	2	Zymo	2.94	0.04	13.0	6.3	1mg gel slice	0.63	n/a
	3	Zymo	3.35	0.07	5.5	-	1mg gel slice	-	n/a
	4	Zymo	-0.73	0.01	2.57	-	1mg gel slice	-	n/a
	5	Zymo	-1.17	0.01	3.61	-	1mg gel slice	-	n/a
CTAB	1	DNeasy	1.92	2.38	337.0	323.0	10 µg DNA	6.4	2
	2	DNeasy	1.92	2.41	370.94	354	10 µg DNA	7.0	n/a
	3	DNeasy	1.93	2.42	338.44	318	10 µg DNA	6.3	n/a
	4	DNeasy	1.90	2.42	300.07	284	10 µg DNA	5.6	n/a
	5	DNeasy	1.90	2.36	273.23	196	10 µg DNA	3.9	n/a
CTAB	1	DNeasy	1.94	2.41	355.91	318	10 µg DNA	6.3	n/a
	2	DNeasy	1.98	2.39	217.26	186	10 µg DNA	3.7	n/a
	3	DNeasy	1.98	2.35	260.41	222	10 µg DNA	4.4	6
CTAB	1	DNeasy	1.92	2.33	381.6	325	10 µg DNA	6.5	7
	2	DNeasy	1.96	2.30	281.26	176	10 µg DNA	3.5	n/a
CTAB	1	DNeasy	1.97	2.35	336.2	324	10 µg DNA	6.4	8
CTAB	1	Genomic-tip 1	1.97	1.48	1272.74	98	100 µg DNA	1.9	n/a
CTAB	2	Genomic-tip 1	2.07	1.91	1815.9	102	100 µg DNA	2.0	3
CTAB	1	Genomic-tip 2	2.06	2.22	1143.4	347	100 µg DNA	6.9	4

* Purification procedures include chloroform:isoamyl alcohol (C:I) extraction, Zymoclean™ Large Fragment DNA Recovery Kit, QIAGEN® DNeasy PowerClean CleanUp Kit, and the QIAGEN® Genomic-tip 500/G (Genomic-tip 1 and Genomic-tip 2).

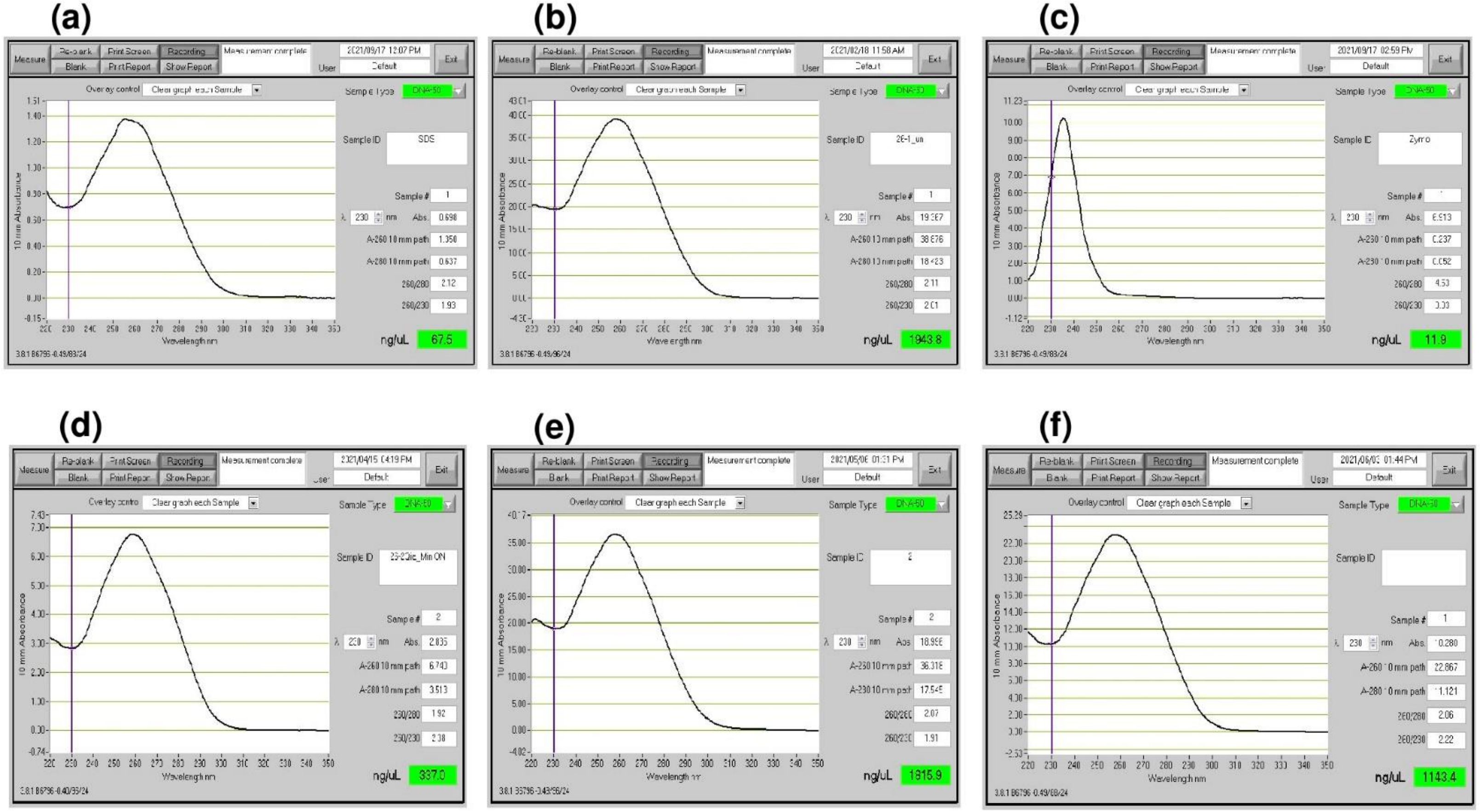

Figure S1: DNA purity and Nanodrop readings from different DNA extraction and purification procedures. (a) DNA extraction using SDS method followed by chloroform:isoamyl alcohol purification. (b) DNA extraction using CTAB protocol with no further purification. (c) CTAB-extracted DNA purified with Zymoclean™ Large Fragment DNA Recovery Kit. (d) CTAB-extracted DNA purified with QIAGEN® DNeasy PowerClean CleanUp Kit. (e) CTAB-extracted DNA purified following Genomic-tip 1 protocol. (f) CTAB-extracted DNA purified following Genomic-tip 2 protocol.
